# Self‐reported diabetes is associated with allocentric spatial processing in the European Prevention of Alzheimer's Dementia Longitudinal Cohort Study

**DOI:** 10.1111/ejn.15821

**Published:** 2022-09-30

**Authors:** Sarah Gregory, Kaj Blennow, Natalie Z. M. Homer, Craig W. Ritchie, Graciela Muniz‐Terrera

**Affiliations:** ^1^ Edinburgh Dementia Prevention, Centre for Clinical Brain Sciences University of Edinburgh Edinburgh UK; ^2^ Institute of Neuroscience and Physiology The Sahlgrenska Academy at the University of Gothenburg Mölndal Sweden; ^3^ Clinical Neurochemistry Laboratory Sahlgrenska University Hospital Mölndal Sweden; ^4^ Mass Spectrometry Core, Edinburgh Clinical Research Facility, Queen's Medical Research Institute University of Edinburgh Edinburgh UK; ^5^ Ohio State University Columbus Ohio USA

**Keywords:** Alzheimer's disease, cohort study, diabetes, prevention, risk factor

## Abstract

Type 2 diabetes is a robust predictor of cognitive impairment. Impairment in allocentric processing may help identify those at increased risk for Alzheimer's disease dementia. The objective of this study was to investigate the performance of participants with and without diabetes on a task of allocentric spatial processing. This was a cross‐sectional secondary data analysis study using baseline data from the European Prevention of Alzheimer's Dementia Longitudinal Cohort Study (EPAD LCS). Participants were aged 50 years and above and were free of dementia at baseline. Participants with no missing data on the variables of interest were included in this study. Our exposure variable was diabetes reported in the medical history. Our primary outcome was the Four Mountains Test (4MT), a novel task of allocentric processing. Covariates included demographics (age, sex, family history of dementia and years of education), *APOEε4* carrier status, cognitive status (Clinical Dementia Rating scale), cerebrospinal fluid phosphorylated tau and amyloid‐beta 1–42. Of 1324 participants (mean age = 65.95 (±7.45)), 90 had diabetes. Participants with diabetes scored 8.32 (±2.32) on the 4MT compared with 9.24 (±2.60) for participants without diabetes. In a univariate model, diabetes was significantly associated with worse 4MT total scores (*β* = −.92, *p* = .001), remaining significant in a fully adjusted model (*β* = −.64, *p* = .01). Cerebrospinal fluid phosphorylated tau was significantly higher in participants with diabetes compared with those without. Novel cognitive tests, such as the 4MT, may be appropriate to identify early cognitive changes in this high‐risk group. Identifying those at greatest risk for future neurodegeneration is key to prevention efforts.

## Graphical abstract

Allocentric processing, assessed using the Four Mountains Test (4MT), refers to the location of objects in respect to each other. Participants with diabetes in the European Prevention of Alzheimer's Dementia Longitudinal Cohort Study performed significantly worse on the 4MT compared with those without diabetes, as well as having higher phosphorylated tau‐181 and performing more poorly on all cognitive domains include in the Repeatable Battery for the Assessment of Neuropsychological Status.

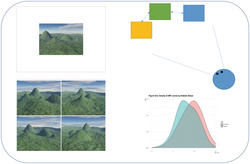

**Abbreviations**
4MT: Four Mountains Test
AD: Alzheimer's disease
Aβ1–42: amyloid‐beta 1–42
BMI: body mass index
CDR: Clinical Dementia Rating
CI: confidence interval
CSF: cerebrospinal fluid
df: degrees of freedom
EPAD LCS: European Prevention of Alzheimer's Dementia Longitudinal Cohort Study
F: female
M: male
MCI: mild cognitive impairment
pTau_181_
: phosphorylated tau‐181
RBANS: Repeatable Battery for the Assessment of Neuropsychological Status
tTau: total tau
*χ* ^2^
: chi‐squared

## 1 INTRODUCTION

The number of people living with dementia is expected to rapidly increase over the coming decades, reaching an estimate of 152 million people by 2050 (Alzheimer's Disease International et al., 2020). Although there are a small number of symptomatic treatments and the recent accelerated approval of aducanumab as a disease‐modifying treatment, all for Alzheimer's disease (AD), attention has shifted in recent years to focus on prevention through identification and management of risk factors, including medical comorbidities. A delay in the onset of dementia even by 2 years has the potential to result in a 19% prevalence in the UK by 2050 (Lewis et al., 2014).

Individuals with type 2 diabetes represent a group at high risk for cognitive impairment, with double the rate of global cognitive decline (Tilvis et al., 2004) and an estimated 1.5 times relative risk for developing AD (Biessels et al., 2006), compared with individuals without diabetes. Furthermore, this relative risk increases to 5.5 times higher risk if the person also has the *APOEε4* allele (Peila et al., 2002) compared with people without type 2 diabetes. Diabetes is one of the important potentially modifiable risk factors discussed by the Lancet Commission group (Livingston et al., 2020). Episodic memory and executive function (McCrimmon et al., 2012) are vulnerable to decline in this patient population. Moreover, the high frequency of type 2 diabetes in many communities means that it is a priority target for population‐level brain health public policy (Ritchie et al., 2010). Potential underlying mechanisms associating type 2 diabetes with AD include the disruptive effect of excess insulin on synaptic plasticity (Biessels & Kappelle, 2005), mitochondrial dysfunction (common to both type 2 diabetes and AD) (Correia et al., 2012), glucose hypometabolism (Li, Risacher et al., 2016) and hypoperfusion (Dake et al., 2020). Hyperinsulinaemia may also lead to competition for insulin‐degrading enzyme, which is important for amyloid clearance (Kurochkin et al., 2018). In fact, AD is often referred to as ‘type 3 diabetes’ (Nguyen et al., 2020), although due to high heterogeneity of both diagnoses, no consensus on definite mechanisms linking these diseases has been reached (Salas & De Strooper, 2019).

Identifying cognitive assessments that are sensitive to early changes associated with AD is an important part of prevention planning, both to screen for those at high risk of AD and as outcomes in clinical and research pathways. As there are currently no interventions known to reduce risk of AD for people living with diabetes, future research into this area is important. Identifying those with diabetes at the highest risk for AD will maximise future clinical trial efficacy, and identification of early cognitive impairment is one avenue to explore.

Progressive hippocampal volume loss is one of the hallmarks of AD (van der Flier & Scheltens, 2009). Traditionally, tasks assessing episodic memory have been utilised as cognitive measures of hippocampal function (Mortamais et al., 2017). However, these tasks are often most useful during the overt phase of disease, and there is a need to introduce more targeted tasks that are effective at detecting impairments and changes during the preclinical disease stage. Functional imaging studies have evidenced the involvement of the hippocampus in the brain network recruited for spatial processing (Iaria et al., 2007). The Four Mountains Test (4MT) (Chan et al., 2016) is a novel task designed to assess allocentric spatial processing abilities. Allocentric spatial processing is the ability to encode and recall viewpoint‐invariant relationships between items and mentally manipulate scenes to consider them from different perspectives. The hippocampus is particularly associated with allocentric spatial processing abilities (Fidalgo & Martin, 2016). Studies in a clinical population using the 4MT show impairment on allocentric processing in participants with AD (Bird et al., 2010). The 4MT has also shown power to discriminate participants with mild cognitive impairment (MCI) from cognitive normal controls and between those with and without cerebrospinal fluid (CSF) AD biomarkers (Moodley et al., 2015). Of particular interest are findings from the PREVENT Dementia study, also led by our group, where 4MT performance was significantly associated with dementia risk score, whereby those with a higher risk score had lower 4MT scores (Ritchie et al., 2018). In this study, interestingly, the 4MT was a better predictor of risk than more traditional hippocampal tests of episodic memory, verbal fluency and executive functioning (Ritchie et al., 2018).

### 1.1 Objective

The aim of this study is to investigate the performance of participants with and without diabetes in the European Prevention of Alzheimer's Dementia Longitudinal Cohort Study (EPAD LCS). The EPAD LCS was a pan‐European cohort study designed to investigate risk factors for dementia across a spectrum of disease stages (Ritchie et al., 2016; Solomon et al., 2018). Our hypothesis was that people with diabetes would perform worse on the 4MT compared with those without diabetes.

## 2 METHODS

### 2.1 Consent and ethics

The EPAD LCS was sponsored by the University of Edinburgh, UK. Separate ethical applications were applied for in each country involved in the project with no study activity taking place until ethical opinions and any country‐ and site‐specific additional regulatory requirements were in place. All participants provided written informed consent after reading approved information sheets and discussing the study with a trained and delegated member of the research team. All participants consented to the future use of their data in research studies.

### 2.2 Data

We used the EPAD LCS v.IMI baseline dataset for this analysis, following approval of a data access request (ep‐ad.org/open‐access‐data/overview). The EPAD LCS is registered at www.clinicaltrials.gov Identifier: NCT02804789. The dataset includes data from all participants who consented to join the study. Data used in preparation of this article were obtained from the EPAD LCS dataset v.IMI (doi:10.34688/epadlcs_v.imi_20.10.30). We excluded participants with missing data in the exposure and outcome variables of interest from the analysis. The EPAD LCS is well described elsewhere (Ritchie et al., 2016; Solomon et al., 2018). In summary, it was a cohort study of participants recruited from other research studies and memory clinics throughout Europe, representing the spectrum of risk for AD dementia from healthy controls and preclinical to prodromal AD. There were generally broad inclusion criteria, with participants required to be aged 50 or above, free of dementia at baseline and able to undergo the protocol assessments. The protocol included baseline, 6 month, 1 year, 2 year and 3 year visits. The long‐term goal was for participants to enter into interventional clinical trials, and therefore, participants were required to be in generally good physical health. At baseline and annual visits, participants completed cognitive assessment batteries, medical and lifestyle history interviews and self‐report questionnaires, provided samples (including blood, saliva, urine and CSF) and underwent magnetic resonance imaging. At 6 month visits, participants completed the cognitive assessment battery and brief medical and lifestyle questionnaires. The EPAD LCS ran from 2015 to 2020.

### 2.3 Variables

Participants completed the 4MT during their baseline assessment period. Prior to undertaking the task, participants are provided with instructions and a small number of practice efforts. Briefly, the 4MT involves participants studying an image of four mountains of varying sizes for a period of about 10 s (see Appendix A). The task involves the use of a touch screen electronic tablet. After a short interval (1 s), the participants are then presented with a second screen with four images. One of these images matches the presenting stimulus but is shown from a different point of view, potentially with different lighting or weather conditions depicted. The participant is tasked with selecting which of the four images is the same as the stimulus image. They do so by tapping the image they want to select on the tablet screen. The software automatically records both whether the response was correct and latency of decision making, reducing possible error introduced by human raters (Chan et al., 2016). The 4MT has been developed to target deficits in those brain regions most associated with early AD pathology and, as such, is a useful research tool in identifying early neurodegenerative disease (Ritchie et al., 2017). As the task is a novel research tool, there are no normative scores available, nor data on the clinical meaningful of change or difference between scores. A lower score indicates a worse performance and could indicate incorrect responses, lack of response or a mixture of both across the 15 trials. We extracted baseline 4MT scores and calculated a total score for all participants to act as the outcome variable as, for this research question, we were interested in overall performance.

We extracted history of diabetes from participants' medical history (self‐reported) as the exposure variable of interest. The medical history does not specify whether participants had type 1 or type 2 diabetes. Exclusion criteria for the cohort included poorly controlled diabetes and those unable to fast for blood samples, which reduces the likelihood that participants with type 1 diabetes were enrolled. Participants with poorly controlled diabetes were excluded as the cohort was designed to be ‘trial‐ready’ for Phase II clinical trials, and this is a common exclusion in AD clinical trials. We also extracted height and weight to calculate body mass index (BMI). BMI was then used to classify participants as obese (BMI of 30 kg/m^2^ or above in line with the World Health Organisation categorisation, World Health Organisation, 2019). We also used blood pressure data to categorise participants as hypertensive or normotensive (hypertensive classified as having either a systolic blood pressure of greater than 140 mmHg or a diastolic blood pressure of greater than 90 mmHg).

We extracted covariates of age, sex (both male and female participants included in this analysis), years of education, family history (self‐reported parental history of dementia, any diagnosis), *APOEε4* carrier status (either heterozygous or homozygous) and Clinical Dementia Rating (CDR) score. We also extracted CSF biomarker data for phosphorylated tau‐181 (pTau_181_) and amyloid‐beta 1–42 (Aβ1–42), measured using the fully automated cobas Elecsys® AD portfolio platform (Elecsys® Total ‐Tau CSF (roche.com)) (Bittner et al., 2016; Lifke et al., 2019) for participants where available for an exploratory analysis. Both pTau_181_ and Aβ1–42 were used in the model as continuous measures. As the 4MT is thought to be associated with hippocampal regions, we calculated a mean of the left and right hippocampal volumes and adjusted the values for intracranial volume. Further, we extracted Repeatable Battery for the Assessment of Neuropsychological Status (RBANS) total and index scores (attention, immediate memory, delayed memory and visuo‐constructional) for participants (Karantzoulis et al., 2013). Comparatively to the 4MT, the RBANS measures more global cognitive deterioration across a number of domains (Ritchie et al., 2017).

### 2.4 Statistical analysis

Initially, we identified participants with missing data in the variables of interest and excluded them from our analysis. Next, we calculated descriptive statistics and tested for differences between individuals included in the analyses and those excluded. Then, we fitted univariate and fully adjusted linear regression models to evaluate the association between 4MT scores and diabetes. The adjusted model of 4MT total score included diabetes and covariates of interest (age, sex, years of education, family history, *APOEε4* carrier status and CDR score). These were included in the model simultaneously. Finally, a model that included an interaction term between diabetes and *APOEε4* carrier status was also included to understand the effect of the interaction between diabetes and *APOEε4* carrier status on 4MT total score. An exploratory analysis then investigated the differences between CSF pTau_181_ and Aβ1–42 in the subgroup of participants with data available and further included these data points as additional covariates in the model, alongside the mean normalised hippocampal volume. Analysis was only run with pTau because of the high correlation between pTau_181_ and total tau (tTau) (rho: .98, *p*<.001). To explore associations with and effects of more global cognitive impairment, differences between mean RBANS index scores of those with and without diabetes were analysed. To understand whether any findings were specific to diabetes, or more generally reflective of cognitive impairment patterns associated with vascular risk factors, we repeated mean test score comparisons for obesity and hypertension. All analyses were conducted in R (Version 4.1.0). Participants with missing data in any field were excluded from the analysis.

## 3 RESULTS

### 3.1 Descriptive statistics

The EPAD baseline sample includes *N* = 2096 individuals. We excluded 772 due to 4MT missing data, resulting in an analytical sample that included 1324 participants in the analysis, 90 (6.7%) of whom had diabetes recorded in their medical history. Excluded participants were younger (*t* test, 65.45 (±7.94) years vs. 66.31 (±7.04) years, *t* = −2.75, degrees of freedom [df] = 2317, *p* = .006), had less education (*t* test, 13.98 (±3.83) years vs. 14.64 (±3.69) years, *t* = −4.20, df = 2317, *p* < .001), were less likely to have a family history of dementia (chi‐squared test, 64.8% vs. 70.8%, *χ* ^2^ = 18.66, df = 2, *p* < .001) and were less likely to be an *APOEε4* carrier (chi‐squared test, 34.9% vs. 40.1%, *χ* ^2^ = 214.31, df = 2, *p* < .001). There was no difference in sex or number of people with diabetes between those excluded and included in the analysis.

Participants with diabetes were older (*t* test, 67.83 (±6.84) years vs. 66.20 (±7.06) years, *t* = −2.13, df = 1322, *p* = .03), more likely to be male (chi‐squared test, male [M] = 54.4% vs. female [F] = 41.4%, *χ* ^2^ = 5.84, df = 1, *p* = .02), less likely to have a family history of dementia (chi‐squared test, 57.8% vs. 71.6%, *χ* ^2^ = 7.67, df = 1, *p* = .006) and more likely to score .5 (indicating MCI) on the CDR (chi‐squared test, 35.6% vs. 22.9%, *χ* ^2^ = 7.46, df = 1, *p* = .006). The proportion of individuals with diabetes did not differ between *APOEε4* carriers (chi‐squared test, 48.9% vs. 39.6%, *χ* ^2^ = 2.99, df = 1, *p* = .106) and non‐carriers or years of education (*t* test, 14.29 years vs. 14.64 years, *t* = .87, df = 1322, *p* = .38).

**FIGURE 1:**
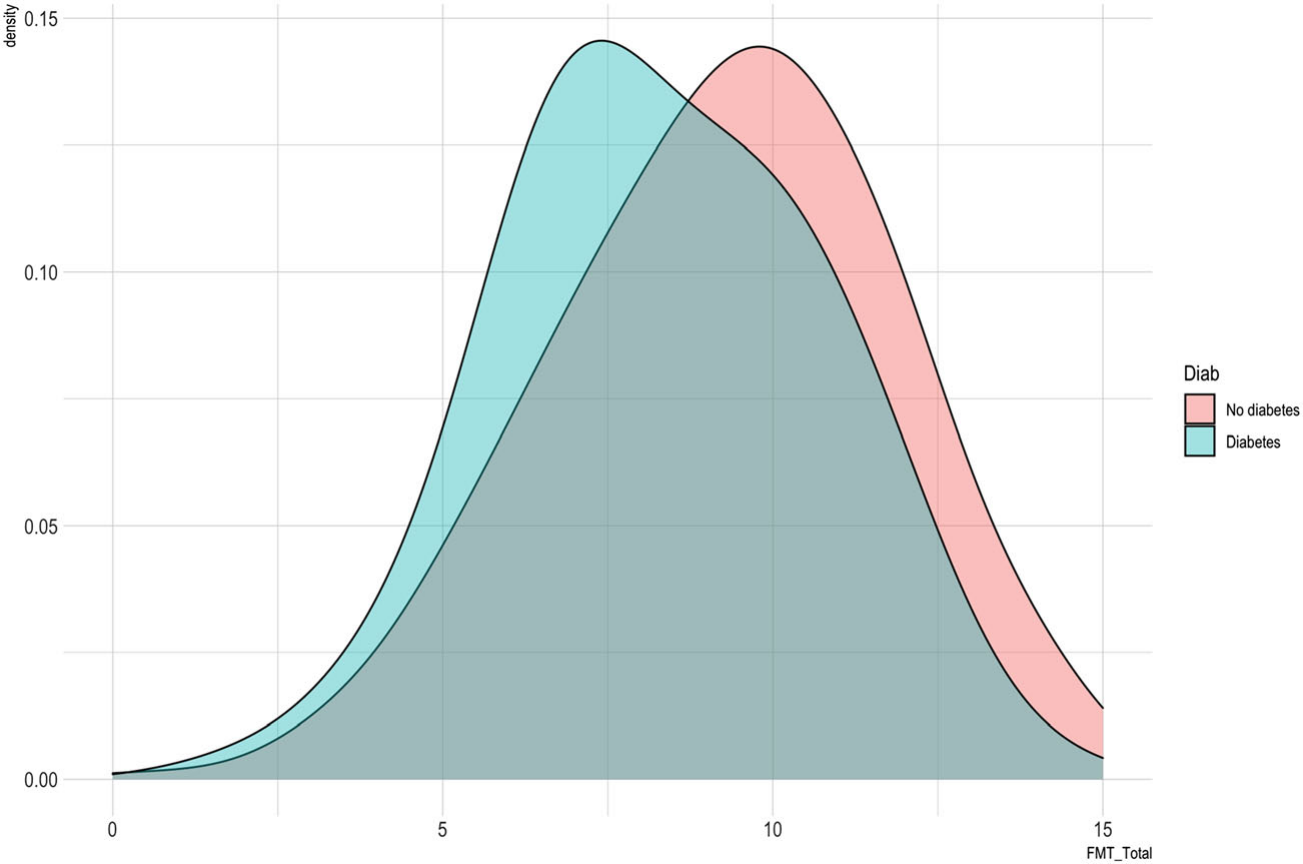
Density of Four Mountains Test (4MT) scores by diabetic status

Descriptive statistics are presented in Table 1. Participants with diabetes had a lower mean total score on the 4MT at baseline visit compared with those without diabetes (8.32 vs. 9.24; range 2–12 vs. 0–15) (see Table 2). Figure 1 shows the density of scores by diabetic status, demonstrating a density shift towards lower scores in the group of participants with diabetes compared with those without.

**TABLE 1:** Descriptive statistics

Variable	With diabetes (*n* = 90)	Without diabetes (*n* = 1234)	*p* value
Age (years), mean (SD)	67.83 (6.84)	66.20 (7.06)	.034*
Sex (male) (*n*, %)	49 (54.4)	511 (41.4)	.021*
*APOEε4* carrier (*n*, %)	44 (48.9)	489 (39.6)	.106
Family history of dementia (*n*, %)	52 (57.8)	883 (71.6)	.006**
Years of education, mean (SD)	14.29 (3.49)	14.64 (3.70)	.383
CDR score (*n*, %)			
0	58 (64.4)	953 (77.2)	.006**
.5	32 (35.6)	281 (22.8)	
Four Mountains Test score, mean (SD); range	8.32 (2.32); 2–12	9.24 (2.60); 0–15	.001**
pTau, mean (SD)^a^	22.48 (14.64)	19.38 (9.29)	.006**
Aβ1–42, mean (SD)^a^	1443.19 (787.64)	1452.09 (745.92)	.92
Hippocampal volume, mean (SD)^b^	5758.99 (722.96)	5700.56 (670.67)	.49

*Note*: Descriptive statistics for participants with (*n* = 90) and without (*n* = 1234) diabetes in the European Prevention of Alzheimer's Dementia Longitudinal Cohort Study (EPAD LCS) v.IMI dataset.

Abbreviations: Aβ1–42, amyloid‐beta 1–42; CDR, Clinical Dementia Rating; pTau, phosphorylated tau.

^a^ Participants with diabetes, *n* = 84; participants without diabetes, *n* = 1146.

^b^ Participants with diabetes, *n* = 80; participants without diabetes, *n* = 1117; hippocampal volume calculated as a mean of left and right hippocampal volume, normalised by intracranial volume.

* *p* < .05.

** *p* < .01.

**TABLE 2:** Associations between diabetes and Four Mountains Test (4MT) scores

Variable	Predictor or covariate	Model 1			Model 2^a^			Model 3^b^		
		*β*	*p*	95% CI	*β*	*p*	95% CI	*β*	*p*	95% CI
Four Mountain Test (baseline visit)	Diabetes	−.92	.001**	−1.47, −.37	−.64	.01*	−1.14, −.13	−.57	.04*	−1.11, −.03
	Sex (female)	—			−.63	<.001***	−.89, −.37	−.69	<.001***	−.98, −.40
	Age				−.13	<.001***	−.14, −.11	−.11	<.001***	−.13, −.09
	Education				.09	<.001***	.05, .12	.09	<.001***	.05, .13
	Family history				−.002	.99	−.29, .28	.09	.58	−.22, .40
	*APOEε4*				−.19	.15	−.45, .07	−.03	.82	−.33, .26
	CDR				−1.73	<.001***	−2.35, −1.11	−.68	<.001***	−1.02, −.34
	pTau				—			−.02	.02*	−.03, −.003
	Aβ1–42							.0001	.14	−.00005, .0003
	Hippocampal volume							.0002	.08	−.0002, .0004

*Note*: Baseline visit 4MT mean scores for participants with (*n* = 90) and without (*n* = 1234) diabetes in the European Prevention of Alzheimer's Dementia Longitudinal Cohort Study (EPAD LCS) v.IMI dataset and outcome of unadjusted (Model 1), fully adjusted (Model 2) and exploratory fully adjusted (Model 3) linear regression models.

Abbreviations: Aβ1–42, amyloid‐beta 1–42; CDR, Clinical Dementia Rating; CI, confidence interval; pTau, phosphorylated tau.

^a^ Fully adjusted model includes diabetes, age, sex, *APOEε4* carrier status, family history of dementia, education and CDR score.

^b^ Exploratory fully adjusted model includes diabetes, age, sex, *APOEε4* carrier status, family history of dementia, education, CDR score, pTau and Aβ1–42.

* *p* < .05.

** *p* < .01.

*** *p* < .001.

### 3.2 Analytical statistics

The univariate linear regression model (Model 1) confirmed this was a statistically significant difference, with diabetes associated with poorer performance in the 4MT test (*β*: −.92, *p* = .001, 95% confidence interval [CI]: −1.47, −.37). Model 2 was a fully adjusted model including the covariates of age, sex, *APOEε4* carrier status, family history of dementia, education and CDR score. In Model 2, the relationship between diabetes and 4MT total score was slightly attenuated but remained statistically significant (*β*: −.64, *p* = .01, 95% CI: −1.14, −.13). Sex (female, *β*: −.63, *p* < .001, 95% CI: −.89, −.37), age (*β*: −.13, *p* < .001, 95% CI: −.14, −.11), years of education (*β*: .09, *p* < .001, 95% CI: .05, .12) and CDR score (mild, *β*: −1.73, *p* < .001, 95% CI: −2.35, −1.11) were all significantly associated with 4MT total score in this model, whereas there was no significant association with family history (*β*: .09, *p* = .99, 95% CI: −.29, .28) or *APOEε4* carrier status (*β*: −.19, *p* = .15, 95% CI: −.45, .07). Results are presented in Table 2.

We ran an exploratory analysis to test for an interaction between *APOEε4* carrier status and diabetes on the 4MT total score based on previous studies citing additional risk of dementia for participants meeting both criteria. We found no significant interaction between diabetes and *APOEε4* carrier status on 4MT total score, whereas the effect of diabetes on 4MT remained significant (diabetes: *β*: −.99, *p* = .012; *APOEε4* carrier: *β*: −.12, *p* = .42; diabetes:*APOEε4* carrier interaction: *β*: .16, *p* = .78).

**FIGURE 2:**
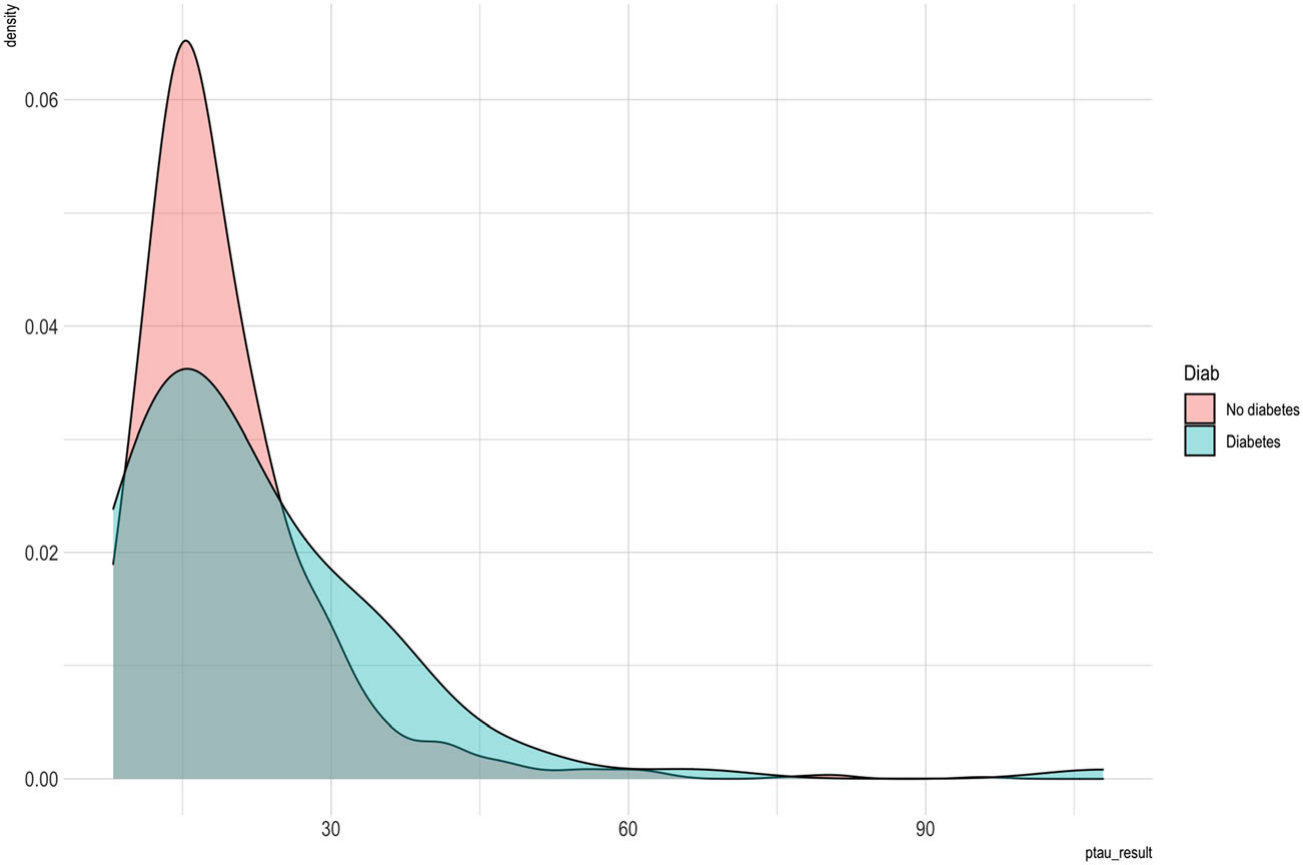
Density of phosphorylated tau‐181 (pTau_181_) concentration by diabetic status

**FIGURE 3:**
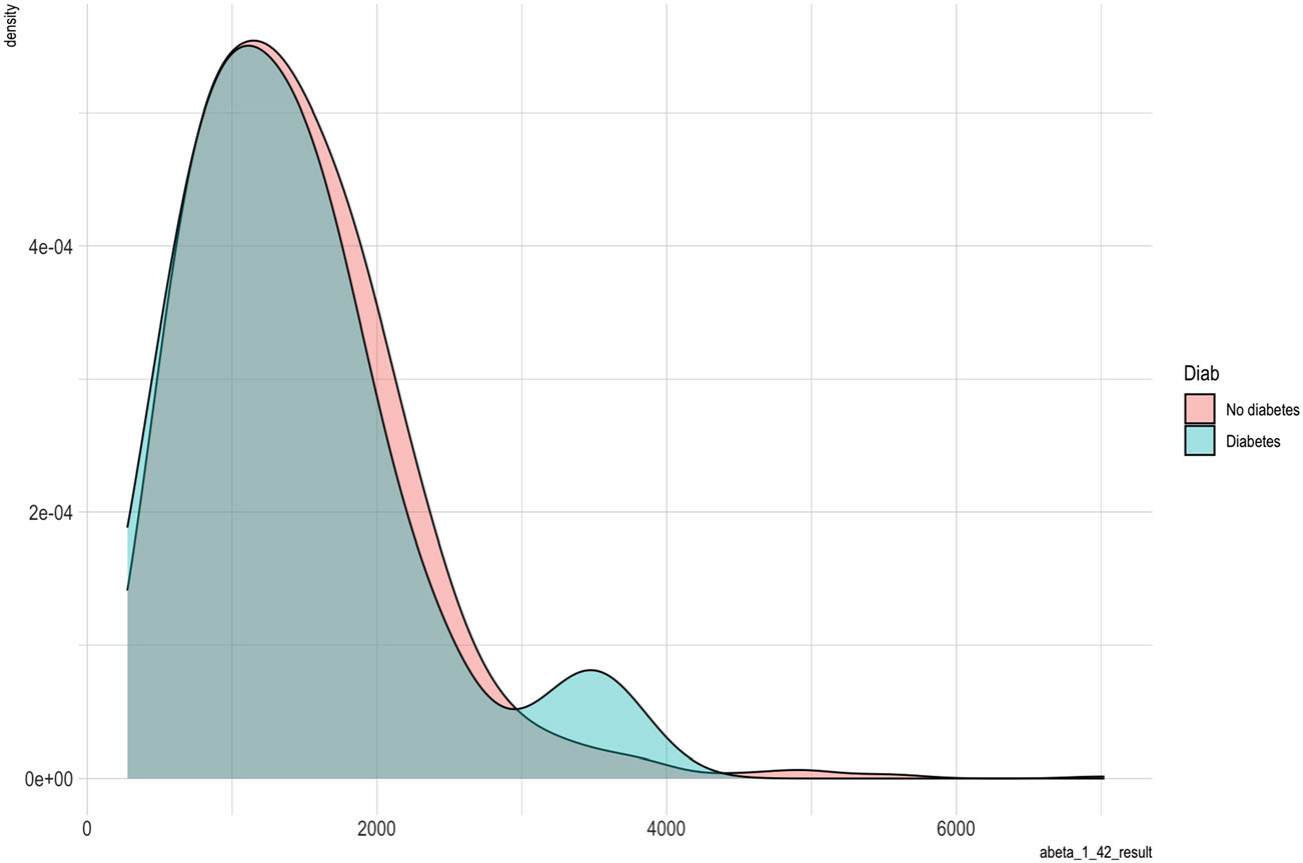
Density of amyloid‐beta 1–42 (Aβ1–42) concentration by diabetic status

A second exploratory analysis investigated the association of the CSF biomarkers (pTau_181_ and Aβ1–42) with diabetes and as covariates in the model. We ran this as an exploratory analysis as there were a number of participants with missing CSF biomarkers and we did not want to reduce the power of our primary analysis. We included 1230 participants in this exploratory analysis. Participants with diabetes had significantly higher CSF pTau_181_ compared with those without diabetes (*t* test, 22.48 (±14.64) vs. 19.38 (±9.29), *t* = −2.78, df = 1215, *p* = .006). There were no significant differences between CSF Aβ1–42 levels in the two groups. Figures 2 and 3 show the density plots of pTau_181_ and Aβ1–42 by diabetes diagnostic group, with the pTau_181_ density suggesting that the significant association with diabetes is driven by those with high levels of pTau_181_ rather than a whole group effect of higher concentrations.

Model 3 was a fully adjusted model including the covariates of age, sex, *APOEε4* carrier status, family history of dementia, education, CDR global score, CSF pTau_181_, CSF Aβ1–42 and mean normalised hippocampal volume. In Model 3, the relationship between diabetes and 4MT total score was largely attenuated but remained statistically significant (*β*: −.57, *p* = .04, 95% CI: −1.11, −.03). Sex (female, *β*: −.69, *p* < .001, 95% CI: −.98, −.40), age (*β*: −.11, *p* < .001, 95% CI: −.13, −.09), years of education (*β*: .09, *p* < .001, 95% CI: .05, .13), CDR global score (*β*: −.68, *p* < .001, 95% CI: −1.02, −.34) and pTau_181_ (*β*: −.02, *p* = .01, 95% CI: −.03, −.003) were all significantly associated with 4MT total score in this model, whereas there was no significant association with family history (*β*: .09, *p* = .58, 95% CI: −.22, .40), *APOEε4* carrier status (*β*: −.03, *p* = .82, 95% CI: −.33, .26), Aβ1–42 (*β*: .0001, *p* = .14, 95% CI: −.00005, .0003) or mean normalised hippocampal volume (*β*: .0002, *p* = .08, 95% CI: −.0002, .0004).

Our final exploratory analysis looked at the performance on RBANS total and index scores between participants with and without diabetes. Participants with diabetes performed significantly worse on the total and all index scores compared with participants without diabetes (total: 97.88 (±15.45) vs. 104.00 (±13.92), *t* = 3.80, df = 1218, *p* < .001; attention: 93.73 (±18.96) vs. 99.87 (16.10), *t* = 3.28, df = 1218, *p* = .001; immediate memory: 99.36 (±17.13) vs. 104.72 (±14.54), *t* = 3.16, df = 1218, *p* = .002; delayed memory: 98.53 (±17.03) vs. 102.83 (±14.86), *t* = 2.49, df = 1218, *p* = .01; visuo‐constructional: 103.28 (±18.15) vs. 108.23 (±15.92), *t* = 2.67, df = 1218, *p* = .008; see Table 3 for details). However, it should be noted that the mean scores in the diabetes group remained within the normal performance range for all test items. Only total and delayed memory index scores were added to the model through a hypothesis‐driven approach that these scores may drive effects seen in 4MT performance. When added to the model, RBANS total index score attenuates the effect of diabetes on 4MT total score (*β* = −.39, *p* = .14, 95% CI: −.91, .13).

**TABLE 3:** Associations between vascular risk factors (diabetes, obesity and hypertension) and cognitive test scores

	Diabetes			Obesity			Hypertension		
	Diabetes	No diabetes	*p*	Obese	Not obese	*p*	Hypertensive	Normotensive	*p*
4MT	8.32 (2.32)	9.24 (2.60)	.001	9.02 (2.61)	9.21 (2.58)	.34	9.01 (2.60)	9.30 (2.57)	.06
RBANS total	97.88 (15.45)	104.00 (139.00)	<.001	101.55 (12.95)	104.00 (14.29)	.02	102.76 (14.27)	104.14 (13.97)	.10
RBANS attention index	93.73 (18.96)	99.87 (14.86)	.001	97.50 (17.23)	99.85 (16.18)	.06	99.68 (15.94)	99.32 (16.66)	.71
RBANS immediate memory index	99.36 (17.13)	104.72 (14.54)	.002	104.81 (13.14)	104.27 (15.08)	.64	102.80 (15.49)	105.39 (14.20)	.003
RBANS delayed memory index	98.53 (17.03)	102.83 (14.86)	.013	102.50 (13.29)	102.55 (15.37)	.97	101.05 (15.71)	103.53 (14.51)	.005
RBANS visuo‐constructional index	103.28 (18.15)	108.23 (15.92)	.008	104.52 (16.86)	108.57 (15.89)	.001	107.37 (16.65)	108.25 (15.76)	.35

*Note*: Baseline cognitive scores across three vascular risk factors for Alzheimer's disease (AD). Data presented for diabetes 4MT (with diabetes [*n* = 90] and without [*n* = 1234]) and RBANS (with diabetes [*n* = 81] and without [*n* = 1139]); obesity (obese [*n* = 201] and not obese [*n* = 1019]); and hypertension (hypertensive [*n* = 486] and normotensive [*n* = 734]) in the European Prevention of Alzheimer's Dementia Longitudinal Cohort Study (EPAD LCS) v.IMI dataset.

Abbreviations: 4MT, Four Mountains Test; RBANS, Repeatable Battery for the Assessment of Neuropsychological Status.

To understand whether the group‐level differences on test performance seen for those with and without diabetes were specific to this diagnostic group or a more general vascular disease pattern, we compared test performance for the 4MT and RBANS for participants with and without obesity and hypertension. Whereas in diabetes, there is a global impairment across all domains, this same pattern is not seen with obesity and hypertension. Obese participants performed significantly worse on RBANS total score as well as the visuo‐constructional index (total: 101.55 (±12.95) vs. 104.00 (±14.29), *t* = 2.25, df = 1218, *p* = .02; visuo‐constructional index: 104.52 (±16.86) vs. 108.57 (±15.89), *t* = 3.27, df = 1218, *p* = .001). Participants with hypertension performed significantly worse on the immediate (102.80 (±15.49) vs. 105.39 (±14.20), *t* = 3.00, df = 1218, *p* = .003) and delayed (101.05 (±15.71) vs. 103.53 (±14.51), *t* = 2.82, df = 1218, *p* = .005) memory indexes compared with normotensive participants. As with the mean RBANS test scores for participants with diabetes, total and index scores for participants with obesity and hypertension were in the normal range. There were no differences in 4MT score or attention index in either obesity or hypertension, suggesting that these results are specific to diabetes. To check for the effect of RBANS attention index scores on the association between diabetes and 4MT, this was added to the fully adjusted model instead of the total RBANS scores and also attenuated the effect of diabetes on 4MT score. Full results are available in Table 3.

## 4 DISCUSSION

This study investigated the association between diabetes and results from the 4MT, a novel task designed to assess allocentric spatial processing abilities. We found that individuals with diabetes had poorer performance on the baseline 4MT as measured by total score in the EPAD LCS v.IMI dataset. This resulted in participants with diabetes scoring an average of .92 points below those without diabetes. The addition of risk variables, hippocampal volume and CSF biomarkers reflecting brain amyloidosis (Aβ1–42) and tau pathology (pTau_181_) did not attenuate this result; however, the addition of the RBANS total index score or attention index score did attenuate this result, suggesting that this is part of a global cognitive deficit in the participants with diabetes. Although there was a global pattern of cognitive impairment seen in people with diabetes across the 4MT and RBANS indexes, only the 4MT and RBANS attention index were specific to diabetes, with no differences seen in these test scores for those with and without obesity or hypertension. This suggests potential value in either or both of these tests for identifying early cognitive changes in the diabetic population. It should also be noted that all of the mean RBANS scores for those with diabetes, as well as other vascular disorders, were within normal limits for the tests, suggesting that these participants may not be being identified in clinical settings as being objectively impaired even though they are performing significantly worse than peers. As we do not have normative scores the 4MT we cannot say at this time if the same would be true were this to be used clinically, and whether those with diabetes would be classified as cognitively normal on this task even though they are performing lower than those without diabetes in our cohort. As the 4MT has been developed to target those areas of earliest tau and amyloid accumulation, rather than the broader cognitive assessment aim of the RBANS, these findings provide initial evidence in support of a more finely tuned selection of tests in at‐risk populations.

Our results align with previous research that showed that individuals with diabetes are more cognitively impaired (McCrimmon et al., 2012). The novel value in this study is using a task of allocentric spatial processing, which has not previously been reported for those with diabetes. The 4MT has previously been associated with MCI, AD and dementia risk score in a cognitively healthy midlife population (Bird et al., 2010; Moodley et al., 2015; Ritchie et al., 2018). The association we found with diabetes in the EPAD LCS v.IMI dataset suggests that this task may be of interest to explore further for suitability as an early marker of cognitive impairment in this at‐risk population. We had anticipated seeing an interaction effect between *APOEε4* carrier status and diabetes as suggested by previous research of the additional risk for developing AD (Peila et al., 2002). We did not see any significant interaction effect and when looking at the contribution of *APOEε4* carrier status in the fully adjusted models, we see no significant association between carrier status and 4MT total score. Similarly to our findings, there was no significant difference on 4MT performance between those with and without a copy of the *APOEε4* allele in the PREVENT Dementia cohort (Ritchie et al., 2018).

Our exploratory analysis found a significant difference in CSF pTau_181_ levels between those with and without diabetes, but no difference in CSF Aβ1–42 levels. The effect of diabetes on 4MT was only slightly attenuated by the addition of these variables and remained significantly associated with 4MT total score. A recent meta‐analysis reviewed the evidence base for CSF tau and amyloid levels in people with diabetes and prediabetes and found that when only looking at those with diabetes, there were significantly higher levels of pTau compared with controls (Lu et al., 2018) consistent with our findings. The reasons for the association with pTau but not amyloid are not well understood. Possible explanations from animal studies include the association between diabetes, advanced glycation end products and tau phosphorylation (Kong et al., 2020), hyperglycaemia‐mediated tau cleavage (Kim et al., 2009) and the activation of glycogen synthase kinase 3β in type 2 diabetes (Kim et al., 2012).

The hippocampus is a region that has a high insulin receptor density and is highly sensitive to metabolic disturbances (Craft & Watson, 2004). Type 2 diabetes has also been associated with reduced hippocampal volumes (den Heijer et al., 2003). Previous neuroimaging studies have found reductions in effective connectivity in the hippocampus in type 2 diabetes and abnormal spontaneous brain activity in the lingual gyrus and calcarine sulcus, which correlates with visual processing and spatial skills (Liu et al., 2020). These studies consistently support the notion that the hippocampus is an area of interest for those living with diabetes and suggest possible mechanisms for the impairment seen on the 4MT, which is sensitive to hippocampal volume loss. Alternatively, a previous study identified a loss of functional connectivity in cerebellar subregions to the cerebral cortex in type 2 diabetes (Zhang et al., 2020), which impacts on the visuospatial networks and leads to impairments on visuospatial tasks (Cui et al., 2017). One theory for these deficits is the modulating impact of insulin levels in these brain regions (Cui et al., 2017). It should be noted that there were no significant hippocampal volume differences between participants with and without diabetes in this study and that the inclusion of this variable in the model did not fully attenuate the association between diabetes and 4MT total score. Future research may consider analysing hippocampal subfields, both for associations with diabetes and 4MT performance, as a more sensitive structural imaging measure early in the AD process.

An important consideration when interpreting these results is whether there are alternative explanations for our findings. The most frequent clinical sequalae of diabetes is diabetic retinopathy (Stitt et al., 2016), a microvascular complication that leads to loss of vision and has an estimated annual incidence of 2.2%–12.7% (Sabanayagam et al., 2019). Despite the common nature of this complication, it frequently goes undiagnosed (Kreft et al., 2018) for a multitude of reasons including lack of awareness, social barriers and attitude to diabetes care (Piyasena et al., 2019). Given this, it is possible that some of the participants with diabetes were living with a degree of diabetic retinopathy, which could have affected their performance on the task.

### 4.1 Limitations

Our study only included diabetes if it was recorded in a participant's medical history. Although some centres confirmed self‐reported medical history through primary care medical record checks, this was not a mandatory part of the protocol. The prevalence of diabetes is relatively low compared with predicated population prevalence in this age group in some of the countries included in the study. The study recruited those in general good health, who were in theory well enough to take part in future clinical trials, and this may be the driving force behind this low prevalence. We do have access to medications taken at the time of the study visit; however, we did not use this to classify participants as type 1 or type 2 diabetes, as it is possible to be prescribed insulin, the main treatment for type 1 diabetes, for type 2 diabetes. Similarly, metformin is an indication of type 2 diabetes; however, as it is used for both prevention and treatment, it cannot give us certainty over type 2 diabetes status. Although considered unlikely, it is possible that a participant did not disclose having diabetes and, therefore, would be included in the control group in this study. It may be more likely that participants were borderline diabetic or prediabetic and were not aware of it. Unfortunately, we do not have access to any biological markers of diabetes at present in the EPAD LCS v.IMI dataset and, as such, it was not possible to have a biologically defined categorisation of diabetes. The EPAD LCS datasets combine participants with both type 1 and type 2 diabetes. Whereas the majority of research to date has focused on the risk conferred by type 2 diabetes (Biessels et al., 2006; Peila et al., 2002; Tilvis et al., 2004), there are also known associations between type 1 diabetes, poor glycaemic control and dementia risk (Lacy et al., 2018).

As the 4MT remains a novel task primarily used in research studies, and no clinical cut‐off points have been derived, it is not yet possible to establish clinical meaningfulness of the nearly one‐point difference found in this study between the groups. However, these results do suggest that the 4MT is sensitive to early cognitive changes in those with diabetes. Although significantly lower test performance was seen across the RBANS tests, there are limitations to this battery that may be important when considering the value of the 4MT in this area. The RBANS is a battery of cognitive tests that requires specialist materials and training to use, taking 20–40 min to complete. In comparison, the 4MT requires no specific staff training (with instructions that participants can read themselves) and takes about 10–15 min to complete, although does currently require an iPad to work. Future development of the 4MT could reduce the time taken to complete even further and make the task more accessible to patients and participants on their own devices, which would allow further testing to really understand the potential value of this task in early disease detection in high‐risk populations, such as those with diabetes.

### 4.2 Future research

Future research will benefit from the study of trajectories of the 4MT and whether they differ by defined diabetes status. Understanding whether those living with diabetes continue to perform more poorly over time compared with those without diabetes will be critical to improve our understanding of the utility of this novel task within this population. A future research question should also explore the role of glycaemic control in moderating any associations with cognition to understand the potential for intervention opportunities, for example, looking at the control and effectiveness of metformin use in prevention and treatment of diabetes. Replication of the analysis in similar cohorts with the same or similar tasks of allocentric spatial processing will be important to confirm the generalisability of the results.

## 5 CONCLUSIONS

Individuals with diabetes perform more poorly on the 4MT, a novel task of allocentric spatial processing, compared with those without diabetes in the EPAD LCS v.IMI dataset. Identification of tasks that can identify early cognitive changes in those at increased risk for dementia is an integral part of a dementia prevention strategy.

## CONFLICTS OF INTEREST

SG previously received salary from the EU/EFPIA grant that was used to fund the data collection (no. 115736) and holds a research grant from the Scottish Neurological Research Fund, which uses the Four Mountains Test as the primary outcome measure. KB has received consulting fees from Abcam, Axon, Biogen, JOMDD/Shimadzu, Julius Clinical, Lilly, MagQu, Novartis, Roche Diagnostics and Siemens Healthineers and is the co‐founder of Brain Biomarker Solutions (Gothenburg, Sweden). CWR has received consulting fees from Biogen, Eisai, MSD, Actinogen, Roche and Eli Lilly and speaker fees from Roche and Eisai. CWR sits on an NIHR data safety monitoring board and is on an advisory board for Roche Diagnostics. CWR is an unpaid chair of the Brain Health Clinic Consortium (sponsored by Biogen), unpaid chair of the Scottish Dementia Research Consortium and director of Brain Health Scotland. NZMH and GMT have no conflicts of interest to disclose.

## AUTHOR CONTRIBUTIONS

SG designed the research question, completed the data analysis and wrote the manuscript. KB and NH reviewed and edited the manuscript. CWR and GMT supervised the research question design, inputted the data analysis and reviewed and edited the manuscript.

### PEER REVIEW

The peer review history for this article is available at https://publons.com/publon/10.1111/ejn.15821.

## Data Availability

Data from the EPAD LCS are openly accessible and available via application at the following website: https://ep-ad.org/open-access-data/overview/. Data used in preparation of this article were obtained from the EPAD LCS dataset v.IMI (doi:10.34688/epadlcs_v.imi_20.10.30).

## APPENDIX A FOUR MOUNTAINS TEST STIMULUS

Participant is shown an image of four mountains of varying sizes for a period of about 10 s. After a brief interval (1 s), the participants are then presented with a second screen with four images. One of these images matches the presenting stimulus but is shown from a different point of view, potentially with different lighting or weather conditions depicted. The participant is tasked with selecting which of the four images is the same as the stimulus image. They do so by tapping the image they want to select on the tablet screen. In this example, the bottom left image matches the first picture.

## References

[ejn15821-bib-0001] Alzheimer's Disease International , Guerchet, M. , Prince, M. , & Prina, M. (2020). Numbers of people with dementia (Full Version, English).

[ejn15821-bib-0002] Biessels, G. J. , & Kappelle, L. J. (2005). Increased risk of Alzheimer's disease in Type II diabetes: Insulin resistance of the brain or insulin‐induced amyloid pathology? Biochemical Society Transactions, 33(5), 1041–1044. 10.1042/BST0331041 16246041

[ejn15821-bib-0003] Biessels, G. J. , Staekenborg, S. , Brunner, E. , Brayne, C. , & Scheltens, P. (2006). Risk of dementia in diabetes mellitus: A systematic review. Lancet Neurology, 5, 64–74. 10.1016/S1474-4422(05)70284-2 16361024

[ejn15821-bib-0004] Bird, C. M. , Chan, D. , Hartley, T. , Pijnenburg, Y. A. , Rossor, M. N. , & Burgess, N. (2010). Topographical short‐term memory differentiates Alzheimer's disease from frontotemporal lobar degeneration. Hippocampus, 20(10), 1154–1169. 10.1002/hipo.20715 19852032

[ejn15821-bib-0005] Bittner, T. , Zetterberg, H. , Teunissen, C. E. , Ostlund, R. E. Jr. , Militello, M. , Andreasson, U. , Hubeek, I. , Gibson, D. , Chu, D. C. , Eichenlaub, U. , Heiss, P. , Kobold, U. , Leinenbach, A. , Madin, K. , Manuilova, E. , Rabe, C. , & Blennow, K. (2016). Technical performance of a novel, fully automated electrochemiluminescence immunoassay for the quantitation of β‐amyloid (1–42) in human cerebrospinal fluid. Alzheimer's & Dementia, 12(5), 517–526. 10.1016/j.jalz.2015.09.009 26555316

[ejn15821-bib-0006] Chan, D. , Gallaher, L. M. , Moodley, K. , Minati, L. , Burgess, N. , & Hartley, T. (2016). The 4 mountains test: A short test of spatial memory with high sensitivity for the diagnosis of pre‐dementia Alzheimer's disease. JoVE, (116), e54454. 10.3791/54454 PMC5092189 27768046

[ejn15821-bib-0007] Correia, S. C. , Santos, R. X. , Carvalho, C. , Cardoso, S. , Candeias, E. , Santos, M. S. , Oliveira, C. R. , & Moreira, P. I. (2012). Insulin signaling, glucose metabolism and mitochondria: Major players in Alzheimer's disease and diabetes interrelation. Brain Research, 1441, 64–78. 10.1016/j.brainres.2011.12.063 22290178

[ejn15821-bib-0008] Craft, S. , & Watson, G. S. (2004). Insulin and neurodegenerative disease: Shared and specific mechanisms. Lancet Neurology, 3(3), 169–178. 10.1016/S1474-4422(04)00681-7 14980532

[ejn15821-bib-0009] Cui, Y. , Liang, X. , Gu, H. , Hu, Y. , Zhao, Z. , Yang, X.‐Y. , Qian, C. , Yang, Y. , & Teng, G.‐J. (2017). Cerebral perfusion alterations in type 2 diabetes and its relation to insulin resistance and cognitive dysfunction. Brain Imaging and Behavior, 11(5), 1248–1257. 10.1007/s11682-016-9583-9 27714551 PMC5653700

[ejn15821-bib-0010] Dake, M. D. , De Marco, M. , Wilkinson, I. , Teh, K. , Mitolo, M. , Remes, A. , Liu, Y. , Pikkarainen, M. , Soininen, H. , & Venneri, A. (2020). Exploring the effect of type 2 diabetes on brain structure and cerebral perfusion in patients with early Alzheimer's disease. Alzheimer's & Dementia, 16(S4), e039401.

[ejn15821-bib-0011] den Heijer, T. , Vermeer, S. E. , van Dijk, E. J. , Prins, N. D. , Koudstaal, P. J. , Hofman, A. , & Breteler, M. M. (2003). Type 2 diabetes and atrophy of medial temporal lobe structures on brain MRI. Diabetologia, 46(12), 1604–1610. 10.1007/s00125-003-1235-0 14595538

[ejn15821-bib-0012] Fidalgo, C. , & Martin, C. B. (2016). The hippocampus contributes to allocentric spatial memory through coherent scene representations. The Journal of Neuroscience, 36(9), 2555–2557. 10.1523/JNEUROSCI.4548-15.2016 26936996 PMC6604870

[ejn15821-bib-0013] Iaria, G. , Chen, J. K. , Guariglia, C. , Ptito, A. , & Petrides, M. (2007). Retrosplenial and hippocampal brain regions in human navigation: Complementary functional contributions to the formation and use of cognitive maps. The European Journal of Neuroscience, 25(3), 890–899. 10.1111/j.1460-9568.2007.05371.x 17298595

[ejn15821-bib-0014] Karantzoulis, S. , Novitski, J. , Gold, M. , & Randolph, C. (2013). The Repeatable Battery for the Assessment of Neuropsychological Status (RBANS): Utility in detection and characterization of mild cognitive impairment due to Alzheimer's disease. Archives of Clinical Neuropsychology, 28(8), 837–844. 10.1093/arclin/act057 23867976

[ejn15821-bib-0015] Kim, B. , Backus, C. , Oh, S. , Hayes, J. M. , & Feldman, E. L. (2009). Increased tau phosphorylation and cleavage in mouse models of type 1 and type 2 diabetes. Endocrinology, 150(12), 5294–5301. 10.1210/en.2009-0695 19819959 PMC2795717

[ejn15821-bib-0016] Kim, D.‐H. , Huh, J.‐W. , Jang, M. , Suh, J.‐H. , Kim, T.‐W. , Park, J.‐S. , & Yoon, S.‐Y. (2012). Sitagliptin increases tau phosphorylation in the hippocampus of rats with type 2 diabetes and in primary neuron cultures. Neurobiology of Disease, 46(1), 52–58. 10.1016/j.nbd.2011.12.043 22245388

[ejn15821-bib-0017] Kong, Y. , Wang, F. , Wang, J. , Liu, C. , Zhou, Y. , Xu, Z. , Zhang, C. , Sun, B. , & Guan, Y. (2020). Pathological mechanisms linking diabetes mellitus and Alzheimer's disease: The receptor for advanced glycation end products (RAGE). Frontiers in Aging Neuroscience, 12, 217. 10.3389/fnagi.2020.00217 32774301 PMC7388912

[ejn15821-bib-0018] Kreft, D. , McGuinness, M. B. , Doblhammer, G. , & Finger, R. P. (2018). Diabetic retinopathy screening in incident diabetes mellitus type 2 in Germany between 2004 and 2013—A prospective cohort study based on health claims data. PLoS ONE, 13(4), e0195426. 10.1371/journal.pone.0195426 29621309 PMC5886553

[ejn15821-bib-0019] Kurochkin, I. V. , Guarnera, E. , & Berezovsky, I. N. (2018). Insulin‐degrading enzyme in the fight against Alzheimer's disease. Trends in Pharmacological Sciences, 39(1), 49–58. 10.1016/j.tips.2017.10.008 29132916

[ejn15821-bib-0020] Lacy, M. E. , Gilsanz, P. , Karter, A. J. , Quesenberry, C. P. , Pletcher, M. J. , & Whitmer, R. A. (2018). Long‐term glycemic control and dementia risk in type 1 diabetes. Diabetes Care, 41(11), 2339–2345. 10.2337/dc18-0073 30181165 PMC6196833

[ejn15821-bib-0021] Lewis, F. , Karlsberg Schaffer, S. , Sussex, J. , O'Neill, P. , & Cockcroft, L. (2014). The trajectory of dementia in the UK—Making a difference. Office of Health Economics Consulting Reports, 2013, 1–55.

[ejn15821-bib-0022] Li, W. , Risacher, S. L. , Huang, E. , Saykin, A. J. , & Alzheimer's Disease Neuroimaging Initiative . (2016). Type 2 diabetes mellitus is associated with brain atrophy and hypometabolism in the ADNI cohort. Neurology, 87(6), 595–600. 10.1212/WNL.0000000000002950 27385744 PMC4977372

[ejn15821-bib-0023] Lifke, V. , Kollmorgen, G. , Manuilova, E. , Oelschlaegel, T. , Hillringhaus, L. , Widmann, M. , von Arnim, C. A. F. , Otto, M. , Christenson, R. H. , Powers, J. L. , Shaw, L. M. , Hansson, O. , Doecke, J. D. , Li, Q. X. , Teunissen, C. , Tumani, H. , & Blennow, K. (2019). Elecsys(®) Total‐Tau and Phospho‐Tau (181P) CSF assays: Analytical performance of the novel, fully automated immunoassays for quantification of tau proteins in human cerebrospinal fluid. Clinical Biochemistry, 72, 30–38. 10.1016/j.clinbiochem.2019.05.005 31129184

[ejn15821-bib-0024] Liu, T. , Bai, Y. , Ma, L. , Ma, X. , Wei, W. , Zhang, J. , Roberts, N. , & Wang, M. (2020). Altered effective connectivity of bilateral hippocampus in type 2 diabetes mellitus. Frontiers in Neuroscience, 14, 657. 10.3389/fnins.2020.00657 32655364 PMC7325692

[ejn15821-bib-0025] Livingston, G. , Huntley, J. , Sommerlad, A. , Ames, D. , Ballard, C. , Banerjee, S. , Brayne, C. , Burns, A. , Cohen‐Mansfield, J. , Cooper, C. , Costafreda, S. G. , Dias, A. , Fox, N. , Gitlin, L. N. , Howard, R. , Kales, H. C. , Kivimäki, M. , Larson, E. B. , Ogunniyi, A. , … Mukadam, N. (2020). Dementia prevention, intervention, and care: 2020 report of the Lancet Commission. The Lancet, 396(10248), 413–446. 10.1016/S0140-6736(20)30367-6 PMC7392084 32738937

[ejn15821-bib-0026] Lu, Y. , Jiang, X. , Liu, S. , & Li, M. (2018). Changes in cerebrospinal fluid tau and β‐amyloid levels in diabetic and prediabetic patients: A meta‐analysis. Frontiers in Aging Neuroscience, 10(271). 10.3389/fnagi.2018.00271 PMC6193181 30364261

[ejn15821-bib-0027] McCrimmon, R. J. , Ryan, C. M. , & Frier, B. M. (2012). Diabetes and cognitive dysfunction. The Lancet, 379(9833), 2291–2299. 10.1016/S0140-6736(12)60360-2 22683129

[ejn15821-bib-0028] Moodley, K. , Minati, L. , Contarino, V. , Prioni, S. , Wood, R. , Cooper, R. , D'Incerti, L. , Tagliavini, F. , & Chan, D. (2015). Diagnostic differentiation of mild cognitive impairment due to Alzheimer's disease using a hippocampus‐dependent test of spatial memory. Hippocampus, 25(8), 939–951. 10.1002/hipo.22417 25605659

[ejn15821-bib-0029] Mortamais, M. , Ash, J. A. , Harrison, J. , Kaye, J. , Kramer, J. , Randolph, C. , Pose, C. , Albala, B. , Ropacki, M. , Ritchie, C. W. , & Ritchie, K. (2017). Detecting cognitive changes in preclinical Alzheimer's disease: A review of its feasibility. Alzheimers Dement, 13(4), 468–492. 10.1016/j.jalz.2016.06.2365 27702618

[ejn15821-bib-0030] Nguyen, T. T. , Ta, Q. T. , Nguyen, T. K. , Nguyen, T. T. , & Van Giau, V. (2020). Type 3 diabetes and its role implications in Alzheimer's disease. International Journal of Molecular Sciences, 21(9), 3165. 10.3390/ijms21093165 32365816 PMC7246646

[ejn15821-bib-0031] Peila, R. , Rodriguez, B. L. , & Launer, L. J. (2002). Type 2 diabetes, APOE gene, and the risk for dementia and related pathologies. Diabetes, 51(4), 1256–1262. 10.2337/diabetes.51.4.1256 11916953

[ejn15821-bib-0032] Piyasena, M. M. P. N. , Murthy, G. V. S. , Yip, J. L. Y. , Gilbert, C. , Zuurmond, M. , Peto, T. , Gordon, I. , Hewage, S. , & Kamalakannan, S. (2019). Systematic review on barriers and enablers for access to diabetic retinopathy screening services in different income settings. PLoS ONE, 14(4), e0198979. 10.1371/journal.pone.0198979 31013274 PMC6478270

[ejn15821-bib-0033] Ritchie, C. W. , Molinuevo, J. L. , Truyen, L. , Satlin, A. , Van der Geyten, S. , & Lovestone, S. (2016). Development of interventions for the secondary prevention of Alzheimer's dementia: The European Prevention of Alzheimer's Dementia (EPAD) project. Lancet Psychiatry, 3(2), 179–186. 10.1016/S2215-0366(15)00454-X 26683239

[ejn15821-bib-0034] Ritchie, K. , Carrière, I. , Howett, D. , Su, L. , Hornberger, M. , O'Brien, J. T. , Ritchie, C. W. , & Chan, D. (2018). Allocentric and egocentric spatial processing in middle‐aged adults at high risk of late‐onset Alzheimer's disease: The PREVENT Dementia study. Journal of Alzheimer's Disease, 65, 885–896. 10.3233/JAD-180432 30103333

[ejn15821-bib-0035] Ritchie, K. , Carrière, I. , Ritchie, C. W. , Berr, C. , Artero, S. , & Ancelin, M. L. (2010). Designing prevention programmes to reduce incidence of dementia: Prospective cohort study of modifiable risk factors. BMJ, 341, c3885. 10.1136/bmj.c3885 20688841 PMC2917002

[ejn15821-bib-0036] Ritchie, K. , Ropacki, M. , Albala, B. , Harrison, J. , Kaye, J. , Kramer, J. , Randolph, C. , & Ritchie, C. W. (2017). Recommended cognitive outcomes in preclinical Alzheimer's disease: Consensus statement from the European Prevention of Alzheimer's Dementia project. Alzheimers Dement, 13(2), 186–195. 10.1016/j.jalz.2016.07.154 27702619

[ejn15821-bib-0037] Sabanayagam, C. , Banu, R. , Chee, M. L. , Lee, R. , Wang, Y. X. , Tan, G. , Jonas, J. B. , Lamoureux, E. L. , Cheng, C.‐Y. , Klein, B. E. K. , Mitchell, P. , Klein, R. , Cheung, C. M. G. , & Wong, T. Y. (2019). Incidence and progression of diabetic retinopathy: A systematic review. The Lancet Diabetes & Endocrinology, 7(2), 140–149. 10.1016/S2213-8587(18)30128-1 30005958

[ejn15821-bib-0038] Salas, I. H. , & De Strooper, B. (2019). Diabetes and Alzheimer's disease: A link not as simple as it seems. Neurochemical Research, 44(6), 1271–1278. 10.1007/s11064-018-2690-9 30523576

[ejn15821-bib-0039] Solomon, A. , Kivipelto, M. , Molinuevo, J. L. , Tom, B. , & Ritchie, C. W. (2018). European Prevention of Alzheimer's Dementia Longitudinal Cohort Study (EPAD LCS): Study protocol. BMJ Open, 8(12), e021017. 10.1136/bmjopen-2017-021017 PMC6318591 30782589

[ejn15821-bib-0040] Stitt, A. W. , Curtis, T. M. , Chen, M. , Medina, R. J. , McKay, G. J. , Jenkins, A. , Gardiner, T. A. , Lyons, T. J. , Hammes, H.‐P. , Simó, R. , & Lois, N. (2016). The progress in understanding and treatment of diabetic retinopathy. Progress in Retinal and Eye Research, 51, 156–186. 10.1016/j.preteyeres.2015.08.001 26297071

[ejn15821-bib-0041] Tilvis, R. S. , Kähönen‐Väre, M. H. , Jolkkonen, J. , Valvanne, J. , Pitkala, K. H. , & Strandberg, T. E. (2004). Predictors of cognitive decline and mortality of aged people over a 10‐year period. The Journals of Gerontology. Series A, Biological Sciences and Medical Sciences, 59(3), 268–274. 10.1093/gerona/59.3.M268 15031312

[ejn15821-bib-0042] van der Flier, W. M. , & Scheltens, P. (2009). Hippocampal volume loss and Alzheimer disease progression. Nature Reviews Neurology, 5(7), 361–362. 10.1038/nrneurol.2009.94 19578342

[ejn15821-bib-0043] World Health Organisation . (2019). Body mass index—BMI. http://www.euro.who.int/en/health-topics/disease-prevention/nutrition/a-healthy-lifestyle/body-mass-index-bmi

[ejn15821-bib-0044] Zhang, D. , Qi, F. , Gao, J. , Yan, X. , Wang, Y. , Tang, M. , Zhe, X. , Cheng, M. , Wang, M. , Xie, Q. , Su, Y. , & Zhang, X. (2020). Altered cerebellar‐cerebral circuits in patients with type 2 diabetes mellitus. Frontiers in Neuroscience, 14(955), 571210. 10.3389/fnins.2020.571210 33071743 PMC7541847

